# The involvement of circulating miR‐146a and miR‐27a in patients with atherosclerotic cardiovascular disease after SARS‐CoV‐2 infection

**DOI:** 10.1002/clc.24274

**Published:** 2024-06-17

**Authors:** Jiahong Zhou, Chao Wei, Guangrong Li, Wenwei He, Miao Song, Xuexue Liu, Jia Feng, Jinbo Liu

**Affiliations:** ^1^ Department of Laboratory Medicine, The Affiliated Hospital of Southwest Medical University, Sichuan Province Engineering Technology Research Center of Molecular Diagnosis of Clinical Diseases Molecular Diagnosis of Clinical Diseases Key Laboratory of Luzhou Luzhou China

**Keywords:** atherosclerotic cardiovascular disease, IL‐6, miR‐146a, miR‐27a, SARS‐CoV‐2 infection

## Abstract

**Background:**

Atherosclerotic cardiovascular disease (ASCVD) is a group of clinical diseases based on pathology of atherosclerosis that is the leading cause of mortality worldwide. There is a bidirectional interaction between ASCVD and severe acute respiratory syndrome coronavirus 2 (SARS‐CoV‐2) infection. Alterations in circulating miRNAs levels are involved in the development of ASCVD in patients infected with SARS‐CoV‐2, however, the correlation between ASCVD co‐infection with SARS‐CoV‐2 and alterations of cardiac‐specific miRNAs is not well understood.

**Hypothesis:**

The circulating miR‐146a and miR‐27a are involved in bidirectional interactions between ASCVD and SARS‐CoV‐2 infections.

**Methods:**

Circulating miR‐146a and miR‐27a levels were measured in serum and PBMCs deriving from ASCVD patients and controls after SARS‐CoV‐2 infection by qRT‐PCR analysis. The levels of neutralizing antibodies‐resistant SARS‐CoV‐2 in human serum was determined by competitive magnetic particle chemiluminescence method. Interleukin (IL)‐6 levels were detected by automatic biochemical analyzer using electrochemiluminescence.

**Results:**

Significant downregulation of circulating miR‐146a and upregulation of miR‐27a in ASCVD patients after infection with SARS‐CoV‐2 compared with controls were observed, among which the alterations were more evident in ASCVD patients comorbid with hyperlipidemia and diabetes mellitus. Consistently, correlation analysis revealed that serum miR‐146a and miR‐27a levels were associated with the levels of lipids and glucose, inflammatory response, and immune function in ASCVD patients. Remarkably, SARS‐CoV‐2 S protein RBD stimulation of PBMCs derived from both ASCVD and controls significantly downregulated miR‐146a, upregulated miR‐27a expression levels, and promoted IL‐6 release in vitro.

**Conclusions:**

The circulating miR‐146a and miR‐27a are involved in metabolism, inflammation, and immune levels in patients with ASCVD after SARS‐CoV‐2 infection, laying the foundation for the development of strategies to prevent the risk of SARS‐CoV‐2 infection in ASCVD patients.

## Graphical abstract

Patients with atherosclerotic cardiovascular disease are more vulnerable to severe acute respiratory syndrome coronavirus 2 (SARS‐CoV‐2) infection and suffer more severe conditions and outcomes, miR‐146a and miR‐27a are involved in bidirectional interactions between ASCVD and SARS‐CoV‐2 infections, and are associated with metabolism, inflammation, and immune levels.

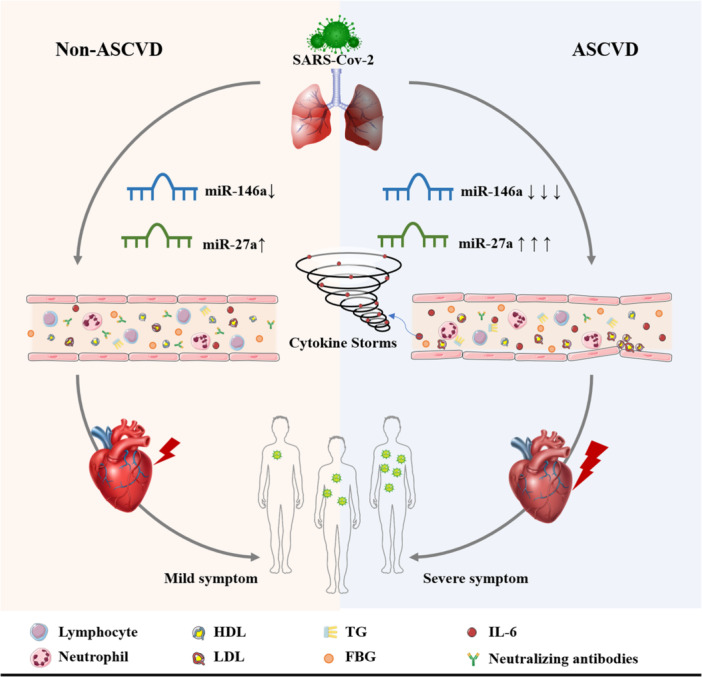

**Abbreviations**
ASCVD: atherosclerotic cardiovascular disease
CAD: coronary atherosclerotic heart disease
COVID‐19: Coronavirus disease‐19
CRP: C‐reactive protein
DM: diabetes mellitus
FASN: fatty acid synthase
FBG: fasting blood glucose
HDL‐C: high‐density lipoproteins cholesterol
HF: heart failure
HL: hyperlipidemia
hs‐TNT: troponin T
HTN: hypertension
ICU: intensive care unit
IL‐6: interleukin‐6
IRAK1: interleukin‐1 receptor‐associated kinase 1
LDL‐C: low‐density lipoproteins cholesterol
MiRNAs: microRNAs
NT‐proBNP: N‐terminal pro B‐type natriuretic peptide
PVD: peripheral vascular disease
RBC: red blood cell
SARS‐CoV‐2: severe acute respiratory syndrome coronavirus 2
TC: total cholesterol
TG: triglycerides
TRAF6: TNF receptor‐associated factor 6

## 1 INTRODUCTION

Atherosclerotic cardiovascular disease (ASCVD), most common cardiovascular disease (CVD), is a group of clinical diseases based on pathology of atherosclerosis (AS) that is the leading cause of mortality worldwide, including coronary atherosclerotic heart disease (CAD), peripheral vascular disease (PVD), stroke and other various CVDs.^1^, ^2^ Hypertension (HTN), diabetes mellitus (DM), hyperlipidemia (HL), and long‐term chronic inflammatory response are the vital risk factors for ASCVD.^2^ Patients with pre‐existing ASCVD are more vulnerable and susceptible to the severe acute respiratory syndrome coronavirus 2 (SARS‐CoV‐2) infection or reinfection, and strongly associated with a poor prognosis.^3^, ^4^ Moreover, SARS‐CoV‐2 infection can lead to new‐onset acute and chronic CVD, such as CAD and heart failure (HF).^5^, ^6^ It was found that CVD (14.5%), HTN (31.2%), DM (10.1%), and malignancy (17.2%) were the most common co‐morbidities among the 138 hospitalized coronavirus disease‐19 (COVID‐19) patients.^7^ Taken together, these data indicate a bidirectional interaction between CVD and SARS‐CoV‐2 infection.

MicroRNAs (miRNAs), 18–25 nucleotide long small noncoding RNA molecules, can regulate gene expression at the posttranscriptional level.^8^ Some heart‐specific miRNAs that play essential roles in maintaining cardiac balance are involved in the development of all stages of AS.^9^, ^10^ In addition, several miRNAs expressed in the cardiovascular system are modified in response to acute cardiac stress or in the long‐term response to chronic injury of the cardiovascular system, and can potentially function as sensitive and specific biomarkers^9^, ^10^; Elgebaly et al showed that circulating miR‐137 and miR‐106b serve as early diagnosis biomarkers of patients with unstable angina^11^ or coronary artery disease.^12^ Interestingly, SARS‐CoV‐2 infection is found to alter the expression of miRNAs related to cardiac function.^13^ However, the correlation between SARS‐CoV‐2 infections and alterations of cardiac‐specific miRNAs is not well understood.

Tiago et al. demonstrated that the expression levels of serum miR‐146 were decreased in patients with AS and were found to be associated with the severe polyvascular AS.^14^ More studies show that miR‐146a is cardioprotective in CVD involving inflammatory responses such as IL‐6, TNF‐α.^15^ Meanwhile, miR‐146a also involved in regulating inflammatory responses induced by SARS‐CoV‐2 infection. In immune cells, miR‐146a is the first miRNA induced after immune activation. Sabbatinelli et al.^16^ revealed elevated serum levels of IL‐6 and reduced levels of miR‐146a in COVID‐19 patients, highlighting the potential role of the imbalance between IL‐6 and miR‐146a in the pathogenesis of COVID‐19. Izzo et al.^17^ summarized the role of miRNAs in cardiovascular complications related to COVID‐19, identifying cardiac‐specific miR‐146a downregulated in COVID‐19 patients. Thus, this implicates a close relationship between miR‐146a and the hyperinflammatory process in SARS‐CoV‐2 and AS. Similarly, it has been demonstrated that the miR‐27a level is altered in patients with AS. Polyakova et al. revealed that miR‐27a is overexpressed in patients with coronary artery disease and is strongly correlated with the severity of coronary AS.^18^ Yao et al. reported that elevated miR‐27a is involved in the progression of coronary artery disease in diabetic patients.^19^ what's more, studies reported that the expression level of miR‐27a was upregulated in severe COVID‐19 patients accompanied by a dramatic increase in IL‐6.^20^ Taken together, it can be suggested that alterations in miR‐146a and miR‐27a after SARS‐CoV‐2 infection may contribute to the development of CVD, including AS. Here, we focus on the potential role of altered levels of circulating miR‐146a and miR‐27a in promoting the progression of ASCVD following SARS‐CoV‐2 infection, and the association with patient clinical parameters.

## 2 MATERIALS AND METHODS

### 2.1 Study subject

The study was conducted on 93 patients with ASCVD and 80 non‐ASCVD controls at Southwest Medical University Affiliated Hospital of China from December 2022 to February 2023. Patients were included in the ASCVD cohort if they had a history of one or more of the following: CAD, peripheral atherosclerotic vascular disease, stroke, and myocardial infarction. Based on the Chinese guidelines for the prevention and treatment of HTN, diabetes and HL, HTN was defined as mean systolic pressure ≥140 mmHg and/or mean diastolic blood pressure ≥90 mmHg; the diabetes was confirmed by (1) fasting blood glucose (FBG) ≥7.0 mmol/L, (2) or random glucose or oral glucose tolerance test (OGTT) 2 h glucose ≥11.1 mmol/L, (3) or glycated hemoglobin (HbA1c) ≥6.5%; and HL was identified by (1) serum total cholesterol (TC) ≥5.72 mmol/L, (2) or elevated blood triglyceride (TG) ≥1.70 mmol/L, (3) or high‐density lipoproteins cholesterol (HDL‐C) ≥3.4 mmol/L, (4) low‐density lipoproteins cholesterol (LDL‐C) <1.0 mmol/L.

All participants enrolled were infected with SARS‐CoV‐2, and have no history of major medical or surgical disease such as malignancy. Peripheral blood samples were collected from subjects for laboratory testing of neutralizing antibodies, miR‐146a, and miR‐27a levels. A standardized records of demographic (gender, age, past history, family history, etc.), clinical, laboratory data were collected from hospital electronic health records after patient inclusion. The study was approved by the Ethics Committee of Southwest Medical University Hospital and informed consent was taken from all patients.

### 2.2 PBMCs isolation and stimulation with SARS‐CoV‐2 S protein RBD in vitro

Peripheral blood samples were collected in EDTA‐K2 tubes from nine patients with ASCVD and nine non‐ASCVD controls who re‐visited our hospital in February 2024 from our previous cohort. PBMCs were isolated from blood by Ficoll‐histopaque‐1077 plus density gradient (GE) centrifugation at 400 g for exactly 30 min at room temperature. PBMCs were washed three times in phosphate‐buffered saline (LOT10771; Sigma‐Aldrich) and subsequently were cultured in complete medium RPMI 1640 (LOTC11875500BT; Gibco) supplemented with 10% fetal bovine serum (LOT C8010‐500Ml; Adamas life) and 100 U/mL penicillin/streptomycin (LotPB180120; Procell) with 5% CO_2_ at 37°C. SARS‐CoV‐2 S protein RBD (Lot264515; MedChemExpress) was used at a final concentration of 0.5 μg/mL to stimulate 1 × 10^7^ PBMCs (*V*_f_  = 1 mL) in 12‐well plates for 24 h. After that, the PBMCs were harvested with Trizol reagent (Lot15596‐026; Invitrogen) for miR‐146a, and miR‐27a assay, and the supernatant was collected for IL‐6 detection.

### 2.3 SARS‐CoV‐2 neutralizing antibodies and IL‐6 detection

The level of neutralizing antibodies against SARS‐CoV‐2 in human serum was determined by Boarsis Axceed 260 fully automated chemiluminescent immunoassay analyzer using a competitive magnetic particle chemiluminescence method equipped with 2019‐nCoV neutralizing antibodies test kit (LOT 2202209008; Bioscience). The results were expressed as S/CO (COI) ratio. IL‐6 levels derived from serum or PBMCs were measured by Cobas 8000 automatic biochemical analyzer using electrochemiluminescence (ECL) equipped with elecsys IL‐6 test kit (LOT05109442190; Roche Diagnostics GmbH).

### 2.4 Determination of miR‐146a and miR‐27a expression level in serum and PBMCs by reverse transcription quantitative real‐time PCR (RT‐qPCR)

Peripheral blood samples (2 mL) were collected in tubes containing EDTA and centrifuged at 10 000 rpm for 10 min at room temperature, then the supernatant was transferred to be stored immediately in RNase free tubes at –80°C until microRNA extraction. MiRNAs extraction were performed using miRcute **serum** separation kit (LOT Y1811; TIANGEN) according to manufacturer's instructions. MiRNAs extraction from PBMCs were similar to that of serum except that lysis was performed using Trizol reagent (Invitrogen, Lot: 15596‐026). The purified miRNAs stored at −80°C until further assessments. Reverse transcription and cDNA preparation of miRNAs were carried out using miRNAs first strand cDNA synthesis kit (LOT # B532451‐0020; Sangon Biotech) according to the manufacturer's instructions and stored at −20°C until qPCR was performed. Quantitative real‐time PCR (qPCR) was carried out using 2xSG Fast qPCR Master Mix (LOT # B639271‐0001; Sangon Biotech). The specific forward primers were designed based on the mature miRNAs sequence, and the sequences were listed as follows: has‐miRNA‐146a‐5p, 5′‐TGAGAACTGAATTCCATGGGTT‐3′; has‐miRNA‐27a‐5p, 5′‐AGGGCTTAGCTGCTTGTGAGCA‐3′; has‐miRNA‐16‐5p, 5′‐TAGCAGCACGTAAATATTGGCG‐3′, while the reverse primers were the universal adaptor sequences supplied by miRNAs first strand cDNA synthesis kit. The qPCR cycling conditions were as follows: 95°C for 3 min, followed by 40 cycles of 95°C for 3 s, 60°C for 30 s in which fluorescence was acquired and detected by real‐time PCR system (SLAN‐96P; HONGSHI), The relative expression levels of the investigated miRNAs were evaluated using the 2^−ΔΔCq^ method.

### 2.5 Statistical analysis

Statistical analysis was performed using IBM SPSS software (version 23.0; IBM), The normality of data distribution was assessed using Shapiro–Wilk test. Discrete variables were presented as frequencies (percentages), continuous variables were presented as mean ± standard deviation follow a normal distribution, and median (interquartile range [IQR]) with nonnormally distributed variables. The comparison between patients with ASCVD and non‐ASCVD controls was made by chi‐square test or Fisher's exact tests for categorical variables, the Mann–Whitney *U* test for continuous variables without a normal distribution. MiR‐146a and miR‐27a expression levels were compared by using the analysis of Mann–Whitney *U* test between two group, and Kruskal–Wallis test among multiple groups following nonnormally distributed variables. The correlation between miRNAs levels and clinical parameters were evaluated by the Spearman's rank correlation coefficients. Alterations in miRNAs and IL‐6 derived from PBMCs were analyzed using independent samples *t*‐tests between ASCVD and controls groups, and paired samples *t*‐tests for pre‐ and post‐S protein of SARS‐CoV‐2 stimulation. *p*‐Value < .05 (two‐tailed) was considered significant.

## 3 RESULTS

### 3.1 Clinical characteristics, laboratory results data of participants

Ninety‐three patients with ASCVD and 80 non‐ASCVD controls were included. The clinical characteristics, outcomes and laboratory results of the participants are presented in Table 1. No significant differences were found for age, gender composition. Patients with ASCVD have more severe clinical manifestations and poorer clinical outcomes. Of the 93 patients, 62 were hospitalized, 15 required oxygenated support, two in‐hospital deaths, and 2–12 developed serious complications, including severe pneumonia, respiratory failure, cardiac arrhythmias, myocarditis and HF. In addition, there is a higher prevalence of metabolic syndrome (MetS), predominantly HL, DM and HTN in patients with ASCVD. Consistently, the levels of lipid, FBG, and the biomarker of inflammation (leukocyte count, neutrophil count, IL‐6, and CRP) and cardiac function (hs‐TNT, NT‐proBNP) were also significantly modified compared to controls group. Interestingly, there was a sharp decrease in the lymphocyte count and percentage relative to controls, and the levels of neutralizing antibodies against SARS‐CoV‐2 were reduced as well.

**Table 1:** Clinical characteristics and laboratory finding of the participants.

Variable	ASCVD (*n* = 93)	Non‐ASCVD (*n* = 80)	*p*‐Value
Clinical characteristics			
Age, years	68 (56–76)	64 (53–72)	.182
Male, *n* (%)	50 (54.35%)	37 (46.3%)	.289
Comorbidities			
Hyperlipidemia	38 (41.3%)	22 (27.5%)	.058
Diabetes mellitus, *n* (%)	60 (65.22%)	5 (6.3%)	<.001
Hypertension, *n* (%)	37 (40.22%)	9 (11.25%)	<.001
Clinical outcomes			
Hospitalization, *n* (%)	62 (66.67%)	3 (3.75%)	<.001
Oxygen/ventilation, *n* (%)	15 (16.13%)	0	–
Clinical complications			
Severe pneumonia	7	0	–
Respiratory failure	10	0	–
Cardiac arrhythmias	7	0	–
Myocarditis	2	0	–
Heart failure	12	0	–
Death, *n* (%)	2 (2.15%)	0	–
Laboratory parameters			
RBC count, 10^12^/L	3.87 (3.28–4.37)	4.60 (4.31–4.89)	<.001
Hemoglobin, g/dL	119 (101–133)	139 (131–145)	<.001
Platelet count, 10^9^/L	213 (160–272)	218 (187–258)	.505
Leukocyte count, 10^9^/L	8.15 (6.12–10.36)	5.21 (4.42–6.04)	<.001
Neutrophil count, 10^9^/L	6.04 (4.07–8.13)	2.96 (2.37–3.57)	<.001
Lymphocyte count, 10^9^/L	1.12 (0.79–1.57)	1.71 (1.39–2.16)	<.001
Lymphocyte percentage (%)	15.2 (9.55–23.20)	33.8 (28.25–38.95)	<.001
TC, mg/dL	4.32 (3.36–5.2)	4.45 (3.94–5.24)	.091
LDL‐C, mg/dL	2.54 (1.97–3.17)	2.49 (2.12–3.18)	.286
HDL‐C, mg/dL	1.16 (0.95–1.41)	1.37 (1.15–1.67)	<.001
TG, mg/dL	1.45 (1.01–1.91)	1.23 (0.88–1.65)	.048
FBG, mmol/L	7.18 (5.40–12.83)	5.06 (4.74–5.72)	<.001
hs‐TNT, µg/L	0.0185 (0.009–0.0455)	0.011 (0.006–0.020)	<.001
NT‐proBNP, ng/L	591 (149–2951)	218 (72–602)	<.001
IL‐6, pg/mL	9.55 (4.04–22.25)	3.9 (1.5–14.2)	<.001
CRP, mg/L	15.5 (6.13–36.95)	11.5 (2.2–34.2)	.014
Calcitonin, ng/mL	0.04 (0.02–0.29)	0.03 (0.02–0.19)	.221
Neutralizing antibodies, S/CO	20.91 (5.73–37.08)	35.93 (21.28–39.26)	<.001

*Note*: Categorical variables are expressed as frequency (percentage) and continuous variables as median (interquartile range). Abbreviations: CRP, C‐reactive protein; FBG, fasting blood glucose; HDL‐C, high‐density lipoproteins cholesterol; hs‐TNT, troponin T; IL‐6, interleukin 6; LDL‐C, low‐density lipoproteins cholesterol; NT‐proBNP, N‐terminal pro B‐type natriuretic peptide; RBC, Red blood cell; TC, total cholesterol; TG, triglycerides.

### 3.2 Expression level of circulating miR‐146a and miR‐27a in patients with ASCVD after SARS‐CoV‐2 infection

**Figure 1:**
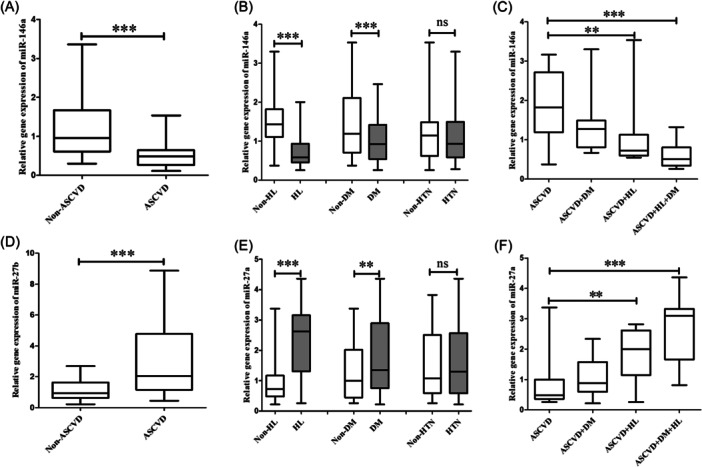
Differential expression level of serum miR‐146a (A–C) and miR‐27a (D–F) levels after SARS‐CoV‐2 infection in ASCVD patients and non‐ASCVD controls, as well as ASCVD patients with or without hyperlipidemia, diabetes, and hypertension. ASCVD, atherosclerotic cardiovascular disease; DM, diabetes; HL, hyperlipidemia; HL, hypertension. **p < .01 and ***p < .001.

In the present study, the expression levels of miR‐146a and miR‐27a between patients with ASCVD and non‐ASCVD controls after SARS‐CoV‐2 infection in their serum were determined by reverse transcription quantitative real‐time PCR. The results showed that the expression level of serum miR‐146a was significantly decreased while miR‐27a was increased in patients with ASCVD compared to the control samples (Figure 1A,D). When grouped the patients by the presence or absence of HL, DM or HTN, further decreased miR‐146a levels and elevated miR‐27a levels were observed in HL and DM, but not the HTN subgroups (Figure 1B,E), however, when patients combined both diabetes and HL, miR‐146a showed a again drop, whereas miR‐27a increases dramatically in serum (Figure 1C,F).

### 3.3 Correlation of miR‐146a and miR‐27a expression level with clinical parameters in ASCVD patients after SARS‐CoV‐2 infection

**Figure 2:**
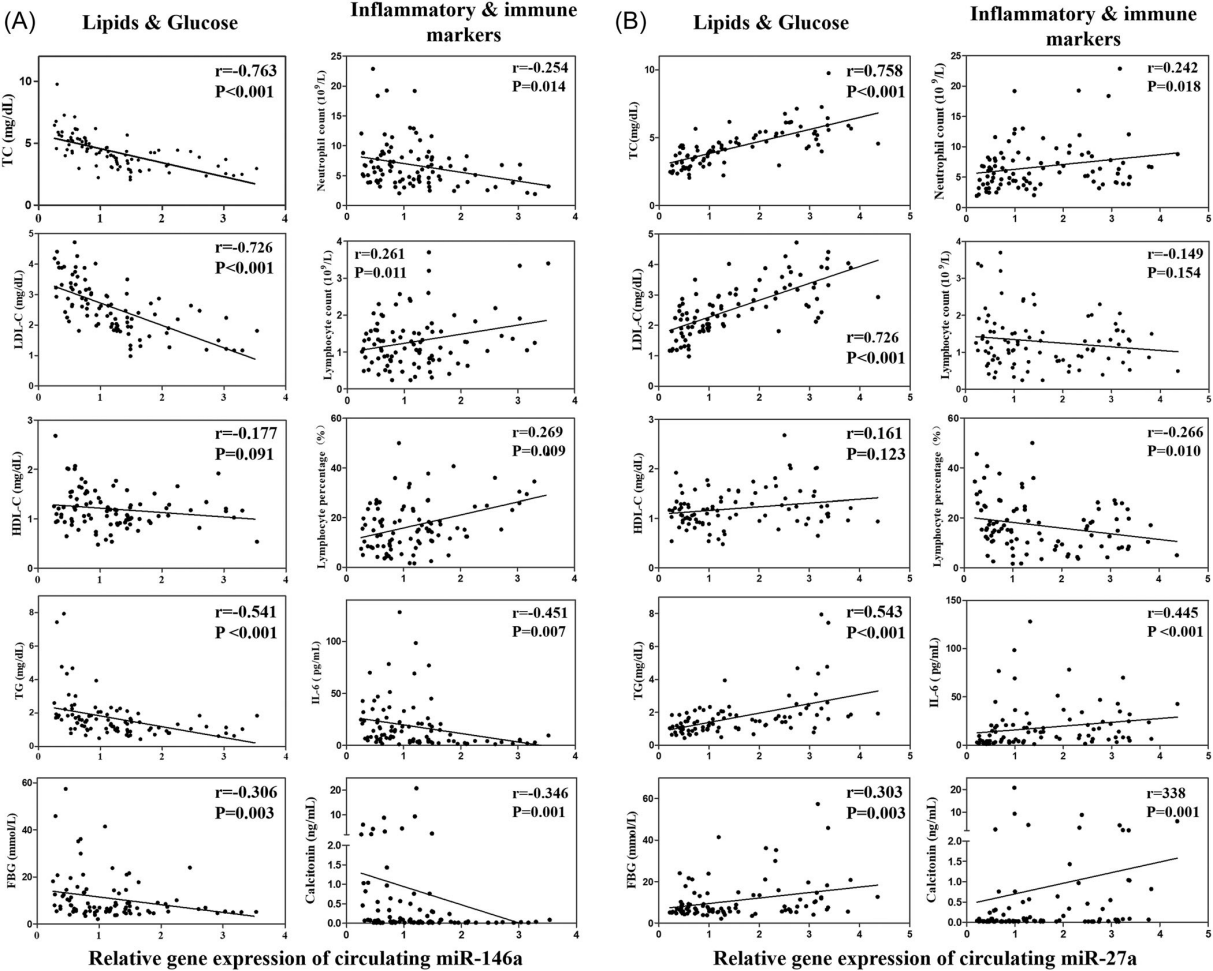
Correlation analysis between miR‐146a (A) and miR‐27a (B) relative expression and clinical parameters of atherosclerotic cardiovascular disease patients infected SARS‐CoV‐2.

The correlation of serum miR‐146a and miR‐27a expression levels with the different clinical parameters was analyzed (Table 2). It was found that the expression level of serum miR‐146a had positive correlations with immunological markers lymphocyte percentage obviously, lymphocyte count and neutralizing antibodies significantly; while it had negative correlations with inflammatory markers neutrophil count, IL‐6, calcitonin remarkably. Interestingly, a significant negative correlation between serum miR‐146a level and lipid levels TC, TG, LDL‐C (but HDL‐C), along with FBG were observed (Figure 2A).

**Table 2:** Correlation of miR‐146a and miR‐27a expression level with clinical parameters.

Variable	miR‐146a		miR‐27a	
	*r*	*p*‐Value	*r*	*p*‐Value
RBC count, 10^12^/L	−.183	.080	.197	.059
Hemoglobin, g/dL	−.195	.061	.209	.054
Platelet count, 10^9^/L	−.021	.839	.033	.737
Leukocyte count, 10^9^/L	−.184	.077	.188	.072
Neutrophil count, 10^9^/L	−.254	.014	.242	.018
Lymphocyte count, 10^9^/L	.261	.011	−.149	.154
Lymphocyte percentage (%)	.269	.009	−.266	.010
TC, mg/dL	−.763	<.001	.758	<.001
LDL‐C, mg/dL	−.726	<.001	.726	<.001
HDL‐C, mg/dL	−.177	.091	.161	.123
TG, mg/dL	−.541	<.001	.543	<.001
FBG, mmol/L	−.306	.003	.303	.003
hs‐TNT, µg/L	−.075	.519	.075	.514
NT‐proBNP, ng/L	−.030	.778	.024	.819
IL‐6, pg/mL	−.451	<.001	.445	<.001
CRP, mg/L	−.100	.342	−.089	.394
Calcitonin, ng/mL	−.246	.001	.338	.001
Neutralizing antibodies, S/CO	.228	.028	−.152	.146

Abbreviations: CRP, C‐reactive protein; FBG, fasting blood glucose; HDL‐C, high‐density lipoproteins cholesterol; hs‐TNT, troponin T; IL‐6, interleukin 6; LDL‐C, low‐density lipoproteins cholesterol; NT‐proBNP, N‐terminal pro B‐type natriuretic peptide; RBC, Red blood cell; TC, total cholesterol; TG, triglycerides.

The association with inflammation, immunity, and metabolism, to some extent, were also found in miR‐27a expression level. The expression level of serum miR‐27a was significantly positively associated with neutrophil count, calcitonin and negatively with lymphocyte percentage, but poorly with neutrophil count, IL‐6, lymphocyte count and neutralizing antibodies. Notably, the levels of miR‐27a were correlated with TC, TG, LDL‐C, FBG level obviously, and statistically significant with HDL‐C (Figure 2B).

Taken together, these data suggest that miR‐146a and miR‐27a seem to play important roles in the inflammatory response, glycolipid metabolism, and immune levels in ASCVD combined with SARS‐CoV‐2 infection.

### 3.4 Alteration of miR‐146a and miR‐27a expression levels by both SARS‐CoV‐2 infection and ASVCD factors

**Figure 3:**
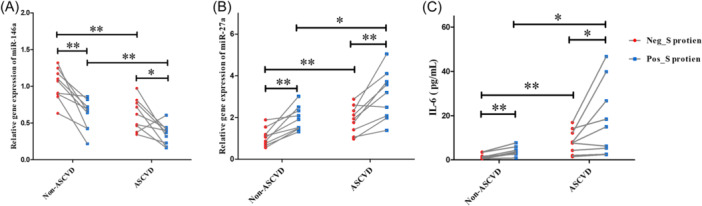
Differential expression levels of miR‐146a (A), miR‐27a (B), and interleukin (IL)‐6 (C) in PBMCs or its supernatant from ASCVD patients and non‐ASCVD controls after stimulating by SARS‐CoV‐2 S protein RBD (0.5 μg/mL) for 24 h. ASCVD, atherosclerotic cardiovascular disease; Neg_S protein: Culturing PBMCs without SARS‐CoV‐2 S protein RBD; Pos_ S protein: PBMCs supplied with SARS‐CoV‐2 S protein RBD for culture. *p < .05, **p < .01.

An important role of miR‐146a and miR‐27a has been observed in patients with ASVCD combined with SARS‐CoV‐2 infection, however, it is unclear whether the regulatory roles of miR‐146a and miR‐27a in ASVCD are affected by SARS‐CoV‐2 infection, therefore, we further obtained PBMCs from 9 follow up ASCVD patients and controls after 1 year of SARS‐CoV‐2 infection, we examined the alterations in the expression of miR‐146a, miR‐27a, and IL‐6 following SARS‐CoV‐2 S protein RBD stimulation in vitro. The results showed that miR‐146a levels were significantly decreased and miR‐27a levels were increased in ASCVD patients compared to controls both with and without SARS‐CoV‐2 S protein RBD stimulation, Interestingly, SARS‐CoV‐2 S protein RBD stimulation would remarkably down‐regulate miR‐146a and up‐regulate miR‐27a expression levels (Figure 3A,B). Consistently, SARS‐CoV‐2 S protein also stimulated the release of the cytokine IL‐6 from PBMCs and had higher levels in patients with ASCVD (Figure 3C).

## 4 DISCUSSION

SARS‐CoV‐2 infection and ASCVD show bidirectional interaction from mechanism to clinical. Patients with underlying disease including CVD, HTN, diabetes, obesity, lymphopenia, and respiratory system disease are more vulnerable to SARS‐CoV‐2 infection and suffer more severe conditions and outcomes.^3^, ^21^ In the current study, ASCVD patients infected with SARS‐CoV‐2 had dramatically higher rates of requiring hospitalization, oxygen support, and experiencing serious complications compared to healthy controls. A cohort study of 1560 COVID‐19 patients showed that having CVD, diabetes, obesity, lymphopenia, dyspnea, and increased AST, ferritin, and CRP were independent predictors for intensive care unit (ICU) admission in patients with COVID‐19.^22^ Consistently, in this study, ASCVD patients had an elevated proportion of comorbid diabetes, significantly higher inflammatory markers IL‐6, CRP, and remarkably lower lymphocyte count, lymphocyte percentage and neutralizing antibodies.

Alterations in circulating miRNAs levels are involved in the development of ASCVD in patients infected with SARS‐CoV‐2.^23^ This study provides the insight into the serum level of miR‐146a and miR‐27a which is potentially involved in the pathogenesis of ASCVD comorbid with SARS‐CoV‐2 infection. We observed significant downregulation of circulating miR‐146a and upregulation of miR‐27a in ASCVD patients after infection with SARS‐CoV‐2 compared with non‐ASCVD controls, among which the alterations were more evident in ASCVD patients comorbid with HL and DM. Consistently, correlation analysis revealed that serum miR‐146a and miR‐27a levels were associated with the levels of lipids and glucose in ASCVD patients. These results support the studies that miR‐146a^14^, ^24^, ^25^ and miR‐27a^26^, ^27^ are involved in the progression and may act as serum biomarkers of ASCVD.

Considerable studies have shown that circulating miR‐146a and miR‐27a are altered in patients with abnormalities of glycolipid metabolism. Findings from the meta‐analysis showed that circulating miR‐146a was downregulated in PBMCs and whole blood samples from patients with type 2 diabetes compared to controls.^28^ Similarly, decreased miR‐146a levels were also observed in obesity^29^, ^30^ and hyperlipidaemia.^31^ Accumulating evidence suggests that miR‐27a is an important regulator of lipid metabolism, which is involved in hepatic lipid deposition, triglyceride synthesis, and lipoprotein uptake, and suppresses the expression of numerous lipid metabolism genes, including fatty acid synthase (FASN), ApoA1, ApoB100, and ApoE3.^32^ Moreover, elevated miR‐27a levels were observed in patients with diabetes^33^ or MetS,^34^ and positively correlated with fasting blood glucose (FBG), triglycerides (TG) levels.^34^ The trend of miR‐146a and miR‐27a alteration in patients with abnormalities of glucose and lipid metabolism is consistent with that of the ASCVD patients combined with DM and HL in the present study. Therefore, this suggests that miR‐146a and miR‐27a play an important role in the common pathway of ASCVD with DM and HL.

It has been shown that both miR‐146a and miR‐27a are involved in the inflammatory response by regulating NF‐κB. Zhou et al.^35^ revealed that miR‐146a inhibits the activation of the NF‐κB pathway by targeting TNF receptor‐associated factor 6 and interleukin 1 receptor‐associated kinase 1 and then induces negative feedback regulation of inflammation,^35^ while miR‐27 is involved in the positive regulation NF‐κB.^36^ Yao et al.^19^ reported that miR‐27a inhibitor block cardiac perivascular fibrosis and restore cardiovascular function by decreasing NF‐κB and TGF‐β signaling during insulin treatment of diabetes. In the present study, significant correlation of miR‐146a and miR‐27a with the IL‐6 that is widely acknowledged to be regulated by NF‐κB, as well as other inflammatory makers were also observed. Furthermore, SARS‐CoV‐2 S protein RBD stimulation of PBMCs derived from both ASCVD and healthy controls significantly downregulated miR‐146a, upregulated miR‐27a expression levels, and promoted cytokine IL‐6 release in vitro. These results suggested that miR‐146a and miR‐27a are involved in the hyperinflammatory response, particularly by affecting IL‐6 levels, both in ASCVD and SARS‐CoV‐2 infection. Thus, we hypothesized that miR‐146a and miR‐27a play important roles in promoting the progression of ASCVD by modulating inflammatory levels, especially after suffering a double whammy of SARS‐CoV‐2 infection.

It is of particular interest to determine the correlation between ASCVD and SARS‐Cov‐2. This study focuses on the relationship between miRNAs, SARS‐CoV‐2, and ASCVD, exploring the potential roles of serum miR‐146a and miR‐27a in ASCVD patients infected with SARS‐CoV‐2 and highlighting the importance of miRNAs in ASCVD combined with DM and HL in SARS‐CoV‐2 infection. However, this study included limited samples and the heterogeneity of retrospective studies unavoidably. In addition, this study lacked studies on the mechanisms of miRNAs involvement in metabolism, inflammation, and immune function. Therefore, we would further explore the relationship between miRNAs and immune function, inflammation, or other biological pathways that may be related to the severity of SARS‐CoV‐2 infection.

## 5 CONCLUSIONS

Taken together, the circulating miR‐146a and miR‐27a are involved in metabolism, inflammation, and immune levels in patients with ASCVD after SARS‐CoV‐2 infection, laying the foundation for the development of strategies to prevent the risk of SARS‐CoV‐2 infection in ASCVD patients.

## AUTHOR CONTRIBUTIONS

*Recruited patients and collected clinical data*: Jiahong Zhou, Wenwei He, Chao Wei, Miao Song, and Xuexue Liu. *Experimental design and operation*: Jiahong Zhou, Chao Wei, and Wenwei He. *Conceptualization and investigation*: Jiahong Zhou, Jinbo Liu, JBL, Jia Feng, and Guangrong Li. *Funding acquisition*: Jinbo Liu. *Supervision*: Miao Song, Xuexue Liu, and Guangrong Li. *Writing—original draft*: Jiahong Zhou and Chao Wei. *Writing—review and editing*: Jinbo Liu, JBL, Jia Feng, and Guangrong Li.

## CONFLICT OF INTEREST STATEMENT

The authors declare no conflict of interest.

## Data Availability

The data that support the findings of this study are available on request from the corresponding author. The data are not publicly available due to privacy or ethical restrictions.
